# Unilateral to Contralateral: A Rare Case of Sequential Obturator Hernias

**DOI:** 10.7759/cureus.78439

**Published:** 2025-02-03

**Authors:** Daniel R Baka, Smeet Madhani, Gregory Withers, Gus Koch, Leon Clarke

**Affiliations:** 1 General Surgery, Philadelphia College of Osteopathic Medicine, Philadelphia, USA; 2 General Surgery, Mercy Fitzgerald Hospital, Darby, USA

**Keywords:** hernia mesh, laparoscopic hernia repair, obstructed obturator hernia, obturator hernias, richter hernia

## Abstract

Obturator hernias are the rarest of all abdominal wall hernias. Multiparous women with a low body mass index (BMI) are at increased risk, and therefore, these hernias are sometimes referred to as “little old lady hernias.” They occur more frequently on the right side than on the left, due to the sigmoid colon protecting the left obturator canal. These hernias are repaired through surgical intervention to prevent complications. In this case report, a thin, elderly, multiparous female patient presented with signs and symptoms of bowel obstruction. Imaging revealed a left-sided obturator hernia, which was repaired laparoscopically with a mesh. Two years later, she returned with similar symptoms, and imaging again revealed an obturator hernia, but this time in the right obturator canal. The hernia was also repaired laparoscopically with a mesh, and the patient experienced no postoperative complications during follow-up.

## Introduction

An obturator hernia is a rare abdominal wall hernia, accounting for less than 1% of all such hernias. However, it is associated with increased mortality compared to other hernias due to its obscure presenting signs and symptoms, which can lead to delayed diagnosis [1,2]. Surgical repair is the mainstay of treatment for such hernias [3]. The optimal timing of surgery depends on the patient’s overall health and comorbidities. Surgery, however, should be performed as soon as possible to prevent bowel strangulation or perforation [4].

This case report describes the clinical trajectory of a thin, elderly female patient who initially presented with a left-sided Richter-type obturator hernia, necessitating laparoscopic repair with a mesh. Two years later, she presented with a distinct right-sided obturator hernia, prompting a similar laparoscopic intervention with mesh placement. In both instances, the patient exhibited signs and symptoms of intestinal obstruction.

## Case presentation

**Figure 1:**
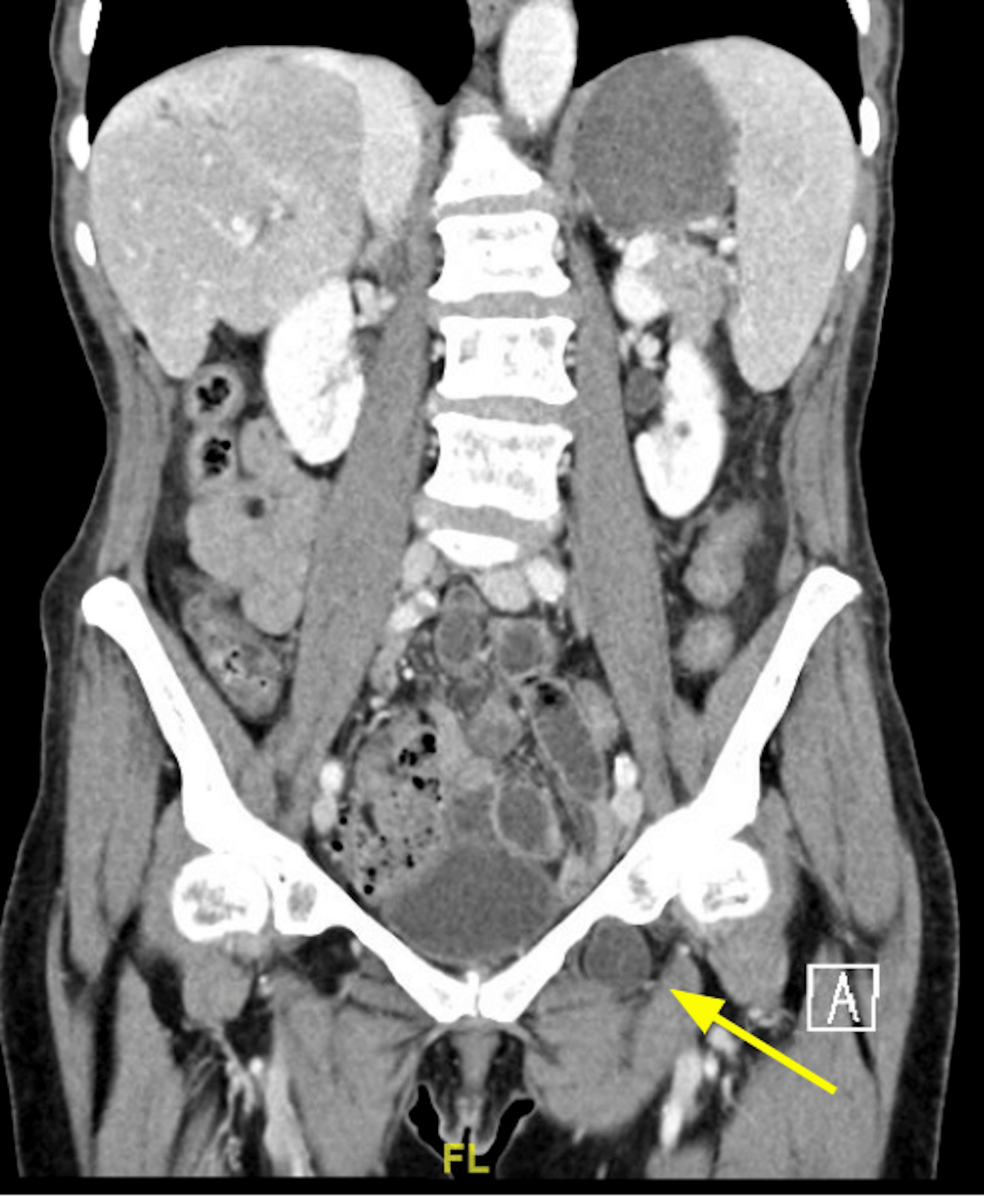
Coronal CT of the abdomen and pelvis demonstrating a portion of the small intestine protruding into the left obturator canal (yellow arrow)

**Figure 2:**
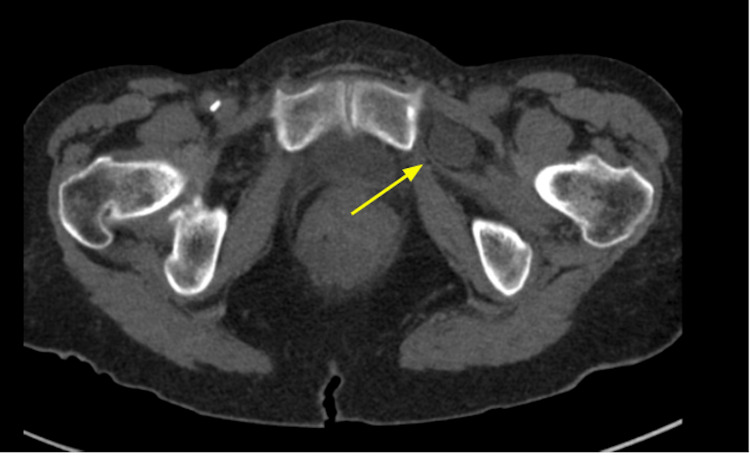
Axial CT demonstrating a left-sided obturator hernia (yellow arrow)

A 77-year-old female patient with a body mass index (BMI) of 19.05 presented to the emergency department (ED) with nausea, abdominal pain, and left anterior thigh pain. The patient’s past medical history included thrombocytopenia and hypertension, and her past surgical history included a cholecystectomy and tubal ligation. Her obstetrical history was significant for multiple pregnancies. The patient had two episodes of emesis upon arrival, but most of her abdominal pain had subsided during her time in the ED. Her vital signs were within normal limits, except for a blood pressure of 154/73 mmHg. A review of her systems was significant for focal weakness and abdominal pain. On physical examination, the abdomen was non distended, without guarding, and no palpable masses were noted in the inguinal region. She had a negative Howship-Romberg sign and her laboratory examination was significant only for a platelet count of 86,000 platelets/mL (reference range: 150,000 - 450,000 platelets/mL). A CT scan showed what appeared to be a left-sided obturator canal hernia with some distention of the bowel proximal to the hernia (Figures 1, 2).

The colon was diffusely thickened. Upon reviewing the CT scan and improvement in physical exam findings, the hernia was considered uncomplicated. The patient was asked to remain fasting or made nil per os (NPO), and laparoscopic repair of the obturator hernia was scheduled for the following morning.

The patient was taken to the operating room. Upon entering the abdomen laparoscopically, a left obturator hernia was noted, containing a loop of the small bowel. The strangulated hernia was reduced and no signs of ischemia were observed. The mesentery was marked with a clip so it could be identified again to check for viability. The peritoneum was dissected, the hernial sac was reduced, and a mesh was placed over the obturator defect. At the time of laparoscopy, no other hernia was observed. The patient’s postoperative course was uncomplicated.

**Figure 3:**
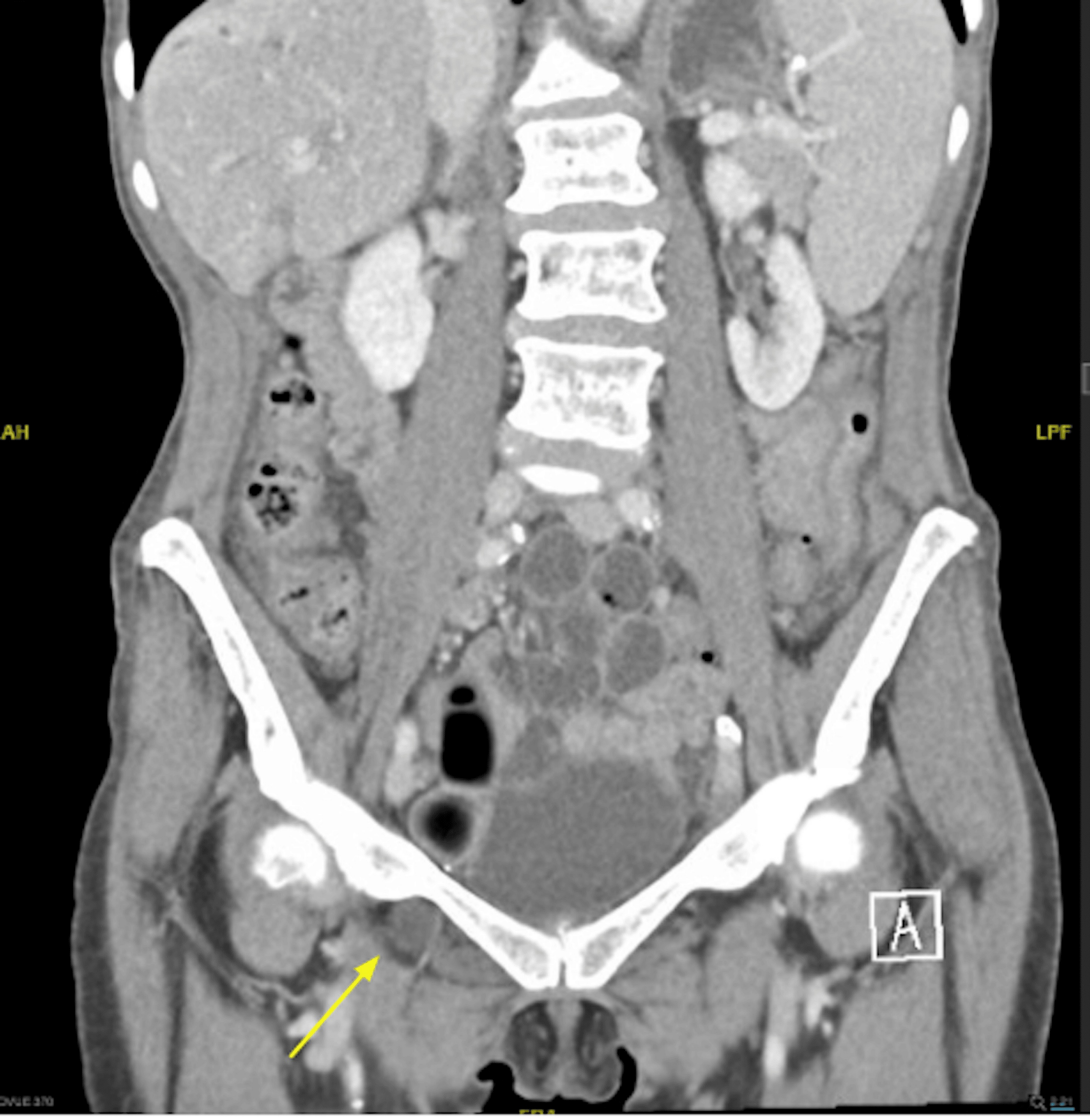
Coronal CT of the abdomen and pelvis demonstrating a portion of the small intestine protruding into the right obturator canal (yellow arrow)

**Figure 4:**
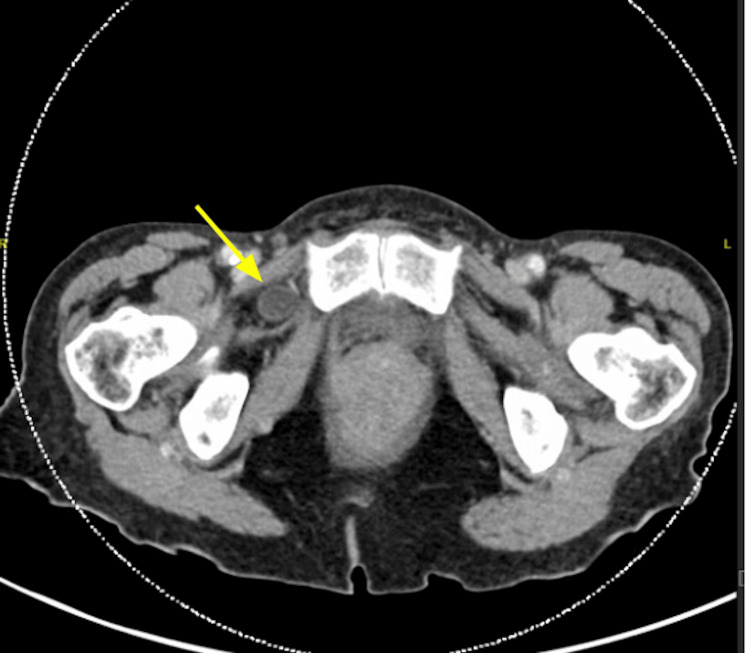
Axial CT demonstrating a right-sided obturator hernia (yellow arrow)

Two years and two months later, the same patient, now 79 years old and with a BMI of 18.05, presented again to the ED complaining of nonbilious, nonbloody vomiting, abdominal pain, and pain in the left leg. Her past medical history remained the same, with the addition of transient ischemic attacks and arthritis. The patient had not undergone any new surgeries since the repair of the left obturator hernia. Her vital signs were within normal limits, except for an elevated blood pressure of 163/81 mmHg. On physical examination, the patient appeared in acute distress and ill. The abdomen was soft, mildly distended in the lower midline, nontender, and tympanic on percussion. The patient again had a negative Howship-Romberg sign. Her complete metabolic panel, complete blood count, urinalysis, and troponins were all within normal limits. A CT scan was performed and showed multiple mildly asymmetric/prominent loops of the small bowel in the pelvis, measuring up to 2.2 cm in caliber. A single loop of fluid-filled, nondilated small bowel was noted as extending into a right obturator hernia (Figures 3, 4).

**Figure 5:**
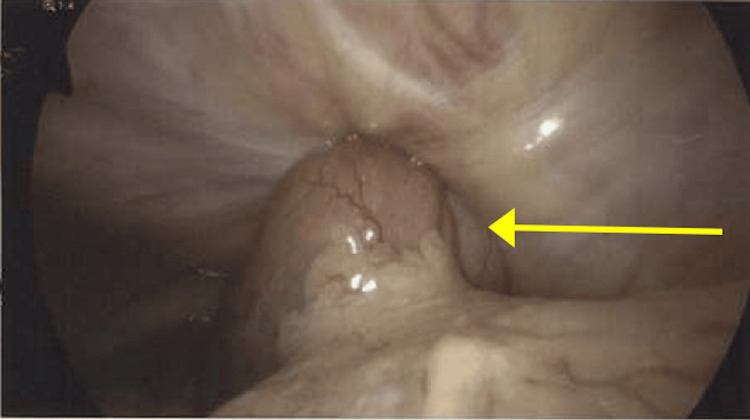
Laparoscopic image of the small bowel herniated into the right obturator canal (yellow arrow)

The patient was promptly taken to the operating room. Upon entering the abdomen with the laparoscope, the abdomen was inspected, and a large reduced left inguinal hernia with the prior mesh was noted, without the evidence of an obturator hernia. A loop of the small bowel was found to be incarcerated within a right obturator hernia (Figure 5).

Using atraumatic graspers, the bowel was gently reduced from the hernia with traction. The incarcerated bowel appeared injected and dark red but immediately began to turn pink with the return of blood flow. No signs of ischemia were noted. The obturator nerve and vessels were carefully dissected and identified, and the right obturator hernial sac was reduced into the peritoneal cavity. A medium-sized piece of mesh was placed and secured to completely cover the right obturator hernial defect.

The patient’s postoperative course was uncomplicated, and she was discharged three days later. At her two- and six-week follow-ups, she was in no pain and had no other complaints.

## Discussion

An obturator hernia is a rare type of abdominal wall hernia with an increased risk of mortality compared to other hernias [5]. Mortality rates in patients with this condition have been reported to be as high as 47.6% [1]. An obturator hernia occurs when the abdominal contents, typically the small bowel, protrude through a weakened or enlarged obturator canal [6].

Understanding the anatomy of the obturator foramen is crucial for grasping the pathology of these hernias. The obturator foramen is a ring-like structure formed by the ischium and pubic bones, covered by the obturator membrane. The internal and external obturator muscles are attached to this membrane. The obturator canal is a small opening in the membrane, which allows the passage of the obturator neurovascular bundle from the abdomen to the medial thigh. This bundle includes the obturator nerve, a branch of the lumbar plexus, which innervates the medial thigh and contributes to clinical signs such as the Howship-Romberg and Hannington-Kiff signs [7,8]. A weakening or an enlargement of the obturator canal can be associated with multiparity. Additionally, the obturator canal is wider and more triangular in women, which explains the nine-fold higher incidence of obturator hernias in women compared to men [5]. Low BMI is another well-documented association [2]. This classic presentation has earned obturator hernias the nickname “little old lady hernias.”

Obturator hernias have been shown to occur more frequently on the right side, likely because the sigmoid colon protects the left obturator canal [9,10]. However, the case presented here involved an initial left-sided obturator hernia, which contrasts with this trend. One retrospective study even found that left-sided obturator hernias were more common [5]. It might be speculated that the patient had bilateral hernias at the initial presentation. However, this is unlikely, as CT imaging revealed unilateral hernias at both presentations, and laparoscopic inspections during both surgeries confirmed no evidence of bilateral hernias. Bilateral obturator hernias do occur and a study of 146 patients found bilaterality in 25.2% of cases [11]. The gradual and insidious progression of this condition makes early detection challenging. Symptoms of obturator hernias can be subtle and nonspecific, including groin pain exacerbated by movement and small bowel obstruction symptoms such as nausea and vomiting [12].

Diagnosing obturator hernias requires a multifaceted approach. A physical examination may occasionally reveal a palpable mass in the groin, although this is rare due to the hernia's location between the pectineus and adductor longus muscles [13]. The Howship-Romberg sign, characterized by medial thigh pain during external rotation, is present in up to 50% of cases [3]. A less well-known but more specific clinical sign is the Hannington-Kiff sign, which consists of a loss of the adductor reflex with preservation of the patellar reflex on the side the hernia occurs [14]. Imaging plays a critical role in diagnosing obturator hernias, with a CT scan being the most reliable diagnostic modality. CT scans not only confirm the presence of the hernia but also identify any associated complications, such as small bowel obstruction [15].

The treatment for an obturator hernia is surgical, as conservative management is not recommended. While no consensus exists on the best surgical approach, a systematic review of 146 patients found that laparoscopic surgery, performed in 30% of cases, significantly reduced morbidity and mortality compared to open repair [11]. Laparoscopic techniques also allow for the detection of occult hernias, enabling their repair and reducing the risk of recurrence [6]. Open hernial repair, when performed, typically involves a low midline incision to locate the hernia, protect the neurovascular structures, expose the obturator ring, and facilitate bowel resection if necessary [10]. Historically, primary repair using sutures was associated with acceptable recurrence rates of less than 10%. However, recent evidence suggests that mesh placement reduces perioperative morbidity rates [10,11]. Further research is needed to resolve ongoing debates regarding the optimal surgical techniques.

## Conclusions

Obturator hernias are a rare subtype of abdominal hernias that can be challenging to diagnose. To improve detection, they should be included in the differential diagnosis for those presenting with bowel obstruction symptoms, particularly thin, elderly female patients. A prompt abdominal and pelvic CT scan should be obtained in such cases. Surgical intervention remains the definitive treatment, with laparoscopic approaches offering reduced morbidity, faster recovery, and the advantage of detecting and repairing occult hernias. If obturator hernias are not identified and surgically treated in a timely manner, bowel obstruction can lead to ischemia or perforation, resulting in complications such as sepsis and death. This case highlights the potential for recurrent and contralateral obturator hernias. Even after successful surgical repair, it is important to follow these patients for an extended period and educate them about risk factors like chronic weight loss or connective tissue weakness. 
